# From bacterial genomics to clinical epidemiology: an interview with Bill Hanage

**DOI:** 10.1186/s12915-018-0588-2

**Published:** 2018-11-01

**Authors:** William P. Hanage

**Affiliations:** Center for Communicable Disease Dynamics, Department of Epidemiology, Harvard TH Chan School of Public Health, Boston, USA

**Keywords:** Epidemiology, Evolution, Genomics, Population genetics, Microbiology, Infectious disease

## Abstract

Bill Hanage is an Associate Professor of Epidemiology at Harvard School of Public Health, where he studies fundamental and applied epidemiology using genomic and evolutionary methods. Bill spoke to us about the different types of selection that determine pathogen populations, asking reviewers to highlight positives of papers, and whether we’re closer to a causal framework for studying the microbiome.

## What are your current research interests?

Pathogen evolution, mostly bacteria, and especially pneumococcus. On the applied side we’re interested in using genome sequences for epidemiology, reconstructing transmission trees [1, 2], and rapidly detecting drug resistance (see this preprint for example [3]). At a more fundamental level we’re into examining the potential of different types of selection to govern the contents of pathogen populations [4]. This is really exciting and our most recent work on it can be read here [5]. Finally, having complained loudly about the lack of one [6], I am edging ever so slowly towards a satisfying causal framework for studying the microbiome. Satisfying to me that is; whether it will satisfy anyone else is a different story.
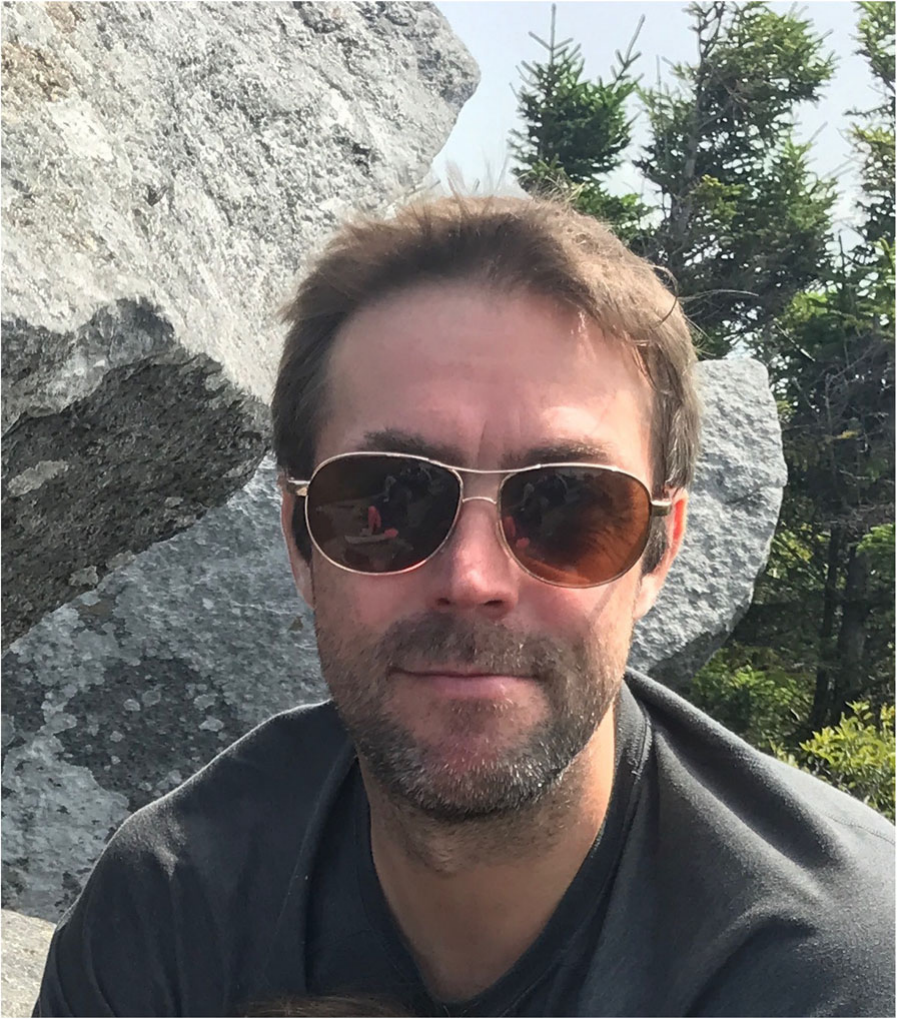


## What are your predictions for the field over the next 5 years?

I think that people using genomes in epidemiology are going to start relating their work to clinical practice, but that this will be less straightforward than might be assumed. For instance, if we had good environmental sampling and detected a drug-resistant bacterium in a hospital ward, what should we do? Shut down the ward, at considerable expense? Resistance genes can be found in non-pathogens too, and the danger will depend on context; for example, whether the ward treats immunocompromised patients.

Speaking about bacterial genomics and evolution more generally I suspect advances in technology are going to move us closer to actual ‘whole genome sequencing’ and away from the ‘high quality draft genome sequencing’ which is what people mostly do now. This might reveal a lot of rapidly accumulating variation in areas that are hard to assemble right now, and greatly expand the list of ‘contingency loci’ [7]. These are areas of the genome where mutation occurs at high enough rates, for example, by strand slippage leading to frame shifts, to produce phenotypic variation in the population. This resulting variation can then be selected, permitting bet hedging strategies to cope with, and rapidly adapt to, a fluctuating environment.

## What motivates you to provide peer review for journals?

The major impetus I have to review papers is that any paper I publish will have needed some nice people to review it (even if they hated it!). So I try to review the number of papers that my own research output would require, reviewing one for each submission. Beyond that sometimes a paper looks just so interesting that I have to review it for reasons of curiosity. However, I have to turn down the vast majority of review requests I get, just because of the sheer volume.

## What changes, if any, would you make to the current system of peer review?

There are obvious problems, and I could write all morning about possible solutions and their benefits and drawbacks. One really simple thing that journals should do is to specifically ask reviewers for positives, which would help steer them towards a rounded view of the work, rather than a laundry list of things that they dislike. My answer to the first question above should make it clear that my lab is increasingly using preprints to get ideas and feedback earlier in the publication process. I am also impressed by attempts to make peer review portable, via things like the Peerage of Science initiative [8]. It would make an enormous difference to have a single central place to which papers are sent, and then journals bid for them. Academics spend a huge amount of time reformatting manuscripts or reviewing papers that have already been peer reviewed by other journals. I’d love to have some of that time back to do science with.

## Have you had any memorably good or bad experiences of peer review, as an author or as a reviewer?

The very first paper I reviewed was lacking a control, which meant all the results might have been the result of contamination with endotoxin. The journal wanted to publish it regardless. I requested to be removed from their list of reviewers and, sure enough, they’ve never asked me again (although to be fair I can’t be sure if that’s because of my request!)

**Twitter:** @BillHanage

**Website:** https://www.hsph.harvard.edu/william-hanage/ and https://c2-d2.github.io/hanage-lab/
